# High Frequencies of Functional Virus-Specific CD4^+^ T Cells in SARS-CoV-2 Subjects With Olfactory and Taste Disorders

**DOI:** 10.3389/fimmu.2021.748881

**Published:** 2021-11-10

**Authors:** Dalila Mele, Anna Calastri, Eugenia Maiorano, Antonella Cerino, Michele Sachs, Barbara Oliviero, Stefania Mantovani, Fausto Baldanti, Raffaele Bruno, Marco Benazzo, Alba Grifoni, Alessandro Sette, Mario U. Mondelli, Stefania Varchetta

**Affiliations:** ^1^ Division of Clinical Immunology and Infectious Diseases, Department of Medicine, Fondazione IRCCS Policlinico San Matteo, Pavia, Italy; ^2^ Division of Otorhinolaryngology, Department of Surgery, Fondazione IRCCS Policlinico San Matteo, Pavia, Italy; ^3^ Division of Infectious Diseases I, Department of Medicine, Fondazione IRCCS Policlinico San Matteo, Pavia, Italy; ^4^ Department of Clinical, Surgical, Diagnostic and Pediatric Sciences, University of Pavia, Pavia, Italy; ^5^ Division of Virology and Microbiology, Fondazione IRCCS Policlinico San Matteo, Pavia, Italy; ^6^ Center for Infectious Disease and Vaccine Research, La Jolla Institute for Immunology, La Jolla, CA, United States; ^7^ Department of Medicine, Division of Infectious Diseases and Global Public Health, University of California, San Diego, La Jolla, CA, United States; ^8^ Department of Internal Medicine and Therapeutics, University of Pavia, Pavia, Italy

**Keywords:** SARS- CoV-2, IFN gamma, virus specific T cells, anosmia, COVID - 19, CD4 and CD8

## Abstract

Olfactory and taste disorders (OTD) are commonly found as presenting symptoms of SARS-CoV-2 infection in patients with clinically mild COVID-19. Virus-specific T cells are thought to play an important role in the clearance of SARS-CoV-2; therefore the study of T cell specific immune responses in patients with mild symptoms may help to understand their possible role in protection from severe disease. We evaluated SARS-CoV-2-specific T cell responses to four different peptide megapools covering all SARS-CoV-2 proteins during the acute phase of the disease in 33 individuals with mild or no other symptom beside OTD and in 22 age-matched patients with severe infection. A control group of 15 outpatients with OTD and consistently negative nasopharyngeal SARS-CoV-2 RNA swabs and virus-specific IgG serology was included in the study. Increased frequencies of virus-specific CD4^+^ and CD8^+^ T cells were found in SARS-CoV-2 positive patients with OTD compared with those with severe COVID-19 and with SARS-CoV-2 negative OTD individuals. Moreover, enhanced CD4^+^ and CD8^+^ T-cell activation induced by SARS-CoV-2 peptides was associated with higher interferon (IFN)γ production. Increased frequencies of Spike (S1/S2)-specific CD4^+^ T cells showing enhanced IFNγ secretion and granzyme B content were associated with serum spike-specific IgG in the OTD group. In conclusion, patients with SARS-CoV-2 induced OTD develop highly functional virus-specific CD4^+^ and CD8^+^ T cells during the symptomatic phase of the disease, suggesting that robust and coordinated T-cell responses provide protection against extension of COVID-19 to the lower respiratory tract.

## Introduction

The spectrum of clinical manifestations of SARS-CoV-2 infection typically spans from cold-like symptoms, cough and fever to severe interstitial pneumonia associated with shortness of breath and respiratory failure requiring assisted ventilation (1, 2). Moreover, anosmia and dysgeusia are recognized as common symptoms of the disease (3–9), and have been added by the Centers for Disease Control and Prevention to the list of symptoms that can occur 2-14 days after exposure to SARS-CoV-2 virus (10). Olfactory and taste disorders (OTD) may occasionally present as the sole manifestation of the disease, whereas OTD caused by other respiratory viruses are associated with inflammation of the respiratory mucosa and mucous discharge (11–14). Anosmia and dysgeusia have been described as the presenting symptom in more than 85% of patients with a mild form of COVID-19 (15, 16), while they are less prevalent in the severe forms of the disease, as indicated by studies reporting a lower rate of OTD in hospitalized patients (5-35%) (17–21). The pathogenesis of anosmia is thought to be related to the high expression of angiotensin-converting enzyme (ACE)-2 receptor, which binds SARS-CoV-2, on the sustentacular cells of the olfactory neuroepithelium, which has been shown to be expressed 200-700 fold more than on nasal or tracheal epithelia (22). In light of this evidence, it has been surmised that, in patients with a stronger upper airway mucosal immunity, SARS-CoV-2 replication may induce a local inflammatory reaction which leads to ear nose and throat (ENT) symptoms and to the generation of an immune response protecting against the development of severe respiratory complications. In contrast, patients with weaker local immunity are thought to more likely develop severe disease (23).

SARS-CoV-2 specific T cells have been identified during convalescence (24–27) and acute infection (27–29). In the acute phase, SARS-CoV-2-specific CD4^+^ T cells have been associated with milder disease (25, 27, 30, 31) suggesting a role for this arm of adaptive immunity in the resolution of the infection. Evidence in support of this hypothesis comes from studies showing low or absent virus-specific CD4^+^ T cells in severe and fatal disease (27, 28, 32). However, detailed knowledge of antiviral immunity in patients with mild or asymptomatic disease is currently limited. Interestingly, the behavior of adaptive immunity in these individuals is controversial, with some studies showing reduced titers of SARS-CoV-2 specific antibodies (33–35), and reduced vigor of T-cell responses (36), whereas more recent studies conducted in asymptomatic individuals have clearly shown that SARS-CoV-2 specific T cells were highly functional and produced sizeable quantities of IFNγ and IL-2 (37).

To gain insight into virus-specific adaptive immune response in SARS-CoV-2 positive subjects with OTD (OTD-CoV-2^pos^), we evaluated SARS-CoV-2 specific T cell responses to peptide megapools covering all SARS-CoV-2 proteins during the acute phase of the disease in individuals with OTD and either mild or no other symptoms and in age-matched patients with severe infection admitted to hospital between February and April 2020. Patients with OTD and consistently negative SARS-CoV-2 serology and nasopharyngeal swab RT-PCR were also included (OTD-CoV-2^neg^).

## Patients

Forty-eight subjects reporting newly-onset OTD were prospectively enrolled over a 30-day period and monitored for four additional weeks. Each individual was visited by an ENT specialist who collected the clinical history to exclude concurrent sinonasal disease or surgery, nasal decongestant or cocaine abuse, neuropsychiatric disorders and head injury. Moreover, the day of symptoms’ onset was recorded in all individuals. Only anterior rhinoscopy was performed because endoscopy was not allowed in these patients because of operator safety issues. None of the patients in the study group had pathological findings after ENT evaluation. Moreover, nasal swabs and serum samples were collected at the time of enrolment (T0) and after one (T7), two (T14) and four weeks (T30). At the same time points, a questionnaire based on the Hyposmia Rating Scale (HRS) (38) and on the Chemotherapy Induced Taste Alteration Scale (CiTAS) (39) was administered at each control to grade hyposmia and hypogeusia. ​ Moreover, smell and taste dysfunctions were graded by a visual analogue scale (VAS) from 0 (worst possible) to 10 (best possible). The findings are reported in **Supplementary Figure 1**. Almost all OTD subjects reported combined perceptive disorders (94.2%), while less than 6% reported only olfactory disorder.

OTD subjects were enrolled in the study a median time of 12 days from symptom onset. Thirty-three (68.7%) were SARS-CoV-2 RNA and/or anti-S1/S2 IgG positive (OTD-CoV-2^pos^) and 15 (31.2%) were consistently SARS-CoV-2 RNA and anti-S1/S2 IgG negative every week for 4 consecutive weeks (OTD-CoV-2^neg^).

Twenty-two patients with radiologically confirmed moderate to severe interstitial pneumonia by chest X-ray and a SARS-CoV-2 RNA positive nasopharyngeal swab or bronchoalveolar lavage were enrolled in the study after a median of 8.5 days from symptom onset. All subjects (100%) were positive for SARS-CoV-2 RNA, 12 (54.5%) had anti-S1/S2 IgG antibody, whereas the remainder (40.9%) were negative for anti-S1/S2 IgG. All patients were hospitalized and required at least a Venturi type of mask for oxygen ventilation (Ventimask^®^ - Flexicare Medical, Ltd). Eleven patients required a ventilation upgrade to Continuous Positive Airway Pressure (C-PAP) with high Positive End Expiratory Pressure (PEEP), three of them were transferred to intensive care unit and intubated. Patient demographics and clinical data are reported in **Supplementary Figure 2** and **Supplementary Table 1**. All the T cell analysis were performed on PBMC obtained at first timepoint (T0) for OTD patients or upon admission to hospital for severe patients.

This study was approved by the Institutional Review Board of Policlinico San Matteo, Italy. All participants signed an informed consent which is conserved in our files. The study complied with the 1975 Declaration of Helsinki.​

## Methods

### PBMC Isolation

PBMC were isolated by standard techniques, resuspended in 90% fetal calf serum (FCS; HyClone, GE Healthcare, South Logan, Utah, USA) + 10% dimethylsulphoxide and stored in liquid nitrogen until use.

Briefly, whole blood was diluted with an equal volume of phosphate-buffered saline (PBS), and diluted blood was layered over Lympholyte (Cedarlane). After centrifugation at 500 × *g* for 30 min at room temperature without the brake applied, the PBMC interface was carefully removed by pipetting and washed with PBS-EDTA by centrifugation at 400 × *g* for 10 min. PBMC pellets were resuspended in PBS containing 2% FCS and washed by centrifugation at 250 × *g* for 10′ at room temperature. Cell numbers were determined by light microscopy count in a Burker chamber. Non-viable cells were identified by staining with trypan blue.

### PBMC Phenotype

Cryopreserved PBMC from patients hospitalized because of COVID-19 severe interstitial pneumonia (severe) and OTD subjects were thawed, washed and rested for 30 min in complete RPMI-1640 medium supplemented with 10% FCS, 2mM L-glutamine and antibiotic antimycotic solution (100 U/ml penicillin, 0.1 μg/ml streptomycin, 0.25 μg/ml amphotericin B (Sigma- Aldrich, St. Louis, MO, USA) (Complete Medium). Subsequently, PBMC were washed and stained for flow cytometric analysis using the following fluorochrome conjugated antibodies: CD8 BV421, CCR7 BV510, PD1 PE-CF594, CD45RA BV650, CD56 BV786, TIM-3 BB515, CD69 PE, CD4 BB700, CD3 APC-H7 (BD Biosciences, San Diego, CA, USA). After fixation and permeabilization (Fixation/Permeabilization Solution Kit; BD Biosciences), cells were stained with anti-Granzyme B Alexa 647 (BD Biosciences). To detect circulating T follicular helper (TFH) cells 1×10^6^ PBMC were stained with the following antibodies: CD3 BV510, CD4 BB700, CD8 BV786, PD1 PE and CXCR5 BV421. The following antibodies were used to identify plasma cells: CD19 BV605, CD27 BB515, CD38 BV421and CD138 BV480. Flow cytometry was performed with a 12-color Celesta (BD Biosciences) instrument and data analyzed with FlowJo 10 software.

### SARS-CoV-2 Specific T Cell Analysis

SARS-CoV-2 specific CD4^+^ and CD8^+^ T cells were analyzed after overnight stimulation with four different peptide megapools (MP), two MP_CD4 (MP_S and MP_R), and two MP_CD8 pools (MP_A and MP_B).

MP_S contained 253 overlapping peptides (15-mers overlapping by 10 amino acids) covering the entire S glycoprotein and can stimulate both CD4^+^ and CD8^+^ T cells. MP_R (R = remainder) contained 221 HLA class II predicted epitopes covering all viral proteins except S, specifically designed to activate CD4^+^ T cells. The two MP_CD8 pools combined contained 628 HLA class I predicted epitopes for the 12 HLA alleles with phenotype frequency ≥6% covering all SARS-CoV-2 proteins, specifically designed to activate CD8^+^ T cells (40).

### *Ex Vivo* Stimulation

After overnight resting, 1×10^6^ PBMC/well were plated in 96 well U-plates in complete medium and stimulated with 1μg/ml of CD4 and CD8 SARS-CoV-2 MPs or equimolar amount of DMSO as negative control. CMV MPs were used as positive controls in some experiments. After 24 hours, supernatants were harvested for IFNγ quantification and SARS-CoV-2 specific T cells were identified by activation-induced markers (AIM), measured as CD134 and CD137 or CD69 and CD137 cell surface co-expression in CD4^+^ and in CD8^+^ T cell subsets, respectively (gating strategy shown in **Supplementary Figure 3**). The following antibodies were used to detect AIM: CD3 BV510, CD4 FITC, CD8 BV786, CD14 BV 605, CD19 BV605, CD134 (OX40) BV421, CD137 (41BB) APC, CD69 PE (BD Biosciences). A LIVE/DEAD^®^ Fixable Near-IR Dead Cell Stain Kit (Thermo Fisher Scientific, Waltham, MA, USA) was used to determine cell viability.

### Serology

SARS-CoV-2-specific antibodies in serum samples were analyzed using Liason SARS-CoV-2 IgG chemiluminescent immunoassay (DiaSorin, Saluggia, Italy) for the quantitative detection of anti-S1 and anti-S2 IgG antibodies, according to the manufacturer’s instructions. Results were given as AU/mL and a cut-off of 13 AU/mL was considered for the definition of positive samples.

### Cytokine Detection

IFNγ secretion was evaluated by DuoSet ELISA (R&D System, MN, USA) in AIM supernatants collected after 24 hours of cell culture in the presence or absence of MPs. AIM samples were freshly thawed, centrifuged (200g for 5 min) and run undiluted according to the manufacturer’s instructions. Serum concentrations of soluble IL-2, -4, -6, -10, -17A, IFNγ and TNFα were determined using the BD Cytometric Bead Array (CBA) Human Th1/Th2/Th17 cytokine kit.​

CXCL13 concentrations were evaluated by means of Human CXCL13/BLC/BCA-1 Quantikine ELISA Kit (R&D System).

### Statistical Analysis

Statistical analysis and graphical presentations were performed using GraphPad Software 8.4 (GraphPad Software Inc, La Jolla, CA). Statistical differences between three groups were assessed by the non-parametric Kruskal-Wallis test followed by Dunn’s multiple comparisons. Non-parametric Wilcoxon matched-pairs signed-rank test was used to compare data within the same group. Mann-Whitney U test was used to compare differences between two groups.

Correlation matrix showing Spearman rank correlation coefficients between paired data was created with Graphpad Prism 8.4.3. The same parameter ordering was used in the two correlograms.

All cytokines reported were measured in pg/mL; e.g., IFNγ MP_R is pg/mL IFNγ in culture supernatants after stimulation of PBMCs with the SARS-CoV-2 CD4-MP_R peptide megapool.

## Results

### Increased Frequency of SARS-CoV-2 Specific CD4 T Cells in OTD-CoV-2^pos^ Subjects

**Figure 1:**
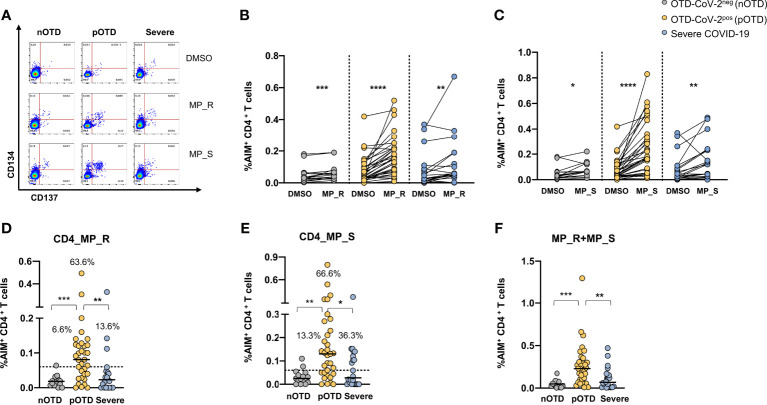
Increased SARS-CoV-2 specific CD4 T cell responses in SARS-CoV-2-infected subjects with OTD. Antigen-specific activation of CD4^+^ T cells was evaluated in PBMC collected from all patients following 24h stimulation with the peptide megapool obtained from the amino acid sequence of the Spike glycoprotein (MP_S), or from the remainder of the SARS-CoV-2 orfeome (MP_R). DMSO was used as a negative control. **(A)** FACS plot examples of CD134 and CD137 co-expression on total CD4^+^ T cells. **(B)** Frequency of AIM+ (CD134^+^ CD137^+^) CD4^+^ T cells obtained by stimulation with MP_S, **(C)** MP_R or DMSO negative control is shown for the 3 different groups. **(D)** Activation of SARS-CoV-2 specific CD4^+^ T cells, in the presence of MP_S and **(E)** MP_R megapools, is shown as percentage AIM^+^ CD4^+^ T cells after subtraction of the vehicle background in the three cohorts. Dotted black lines indicate cut-off values for positivity. **(F)** Combined AIM^+^ CD4^+^ T cells responses to DMSO-subtracted individual peptide megapools. The non-parametric Wilcoxon matched-pairs signed-rank test was used to compare data within the same group. Statistical comparison between three groups of subjects was assessed by the non-parametric Kruskal-Wallis test followed by Dunn’s multiple comparisons. *p < 0.05; **p < 0.01; ***p < 0.001; ****p < 0.0001.

CD4^+^ T cells specific for SARS-CoV-2 were identified by CD134 and CD137 co-expression after stimulations with two megapools: MP_S covering the entire S glycoprotein and MP_R based on CD4^+^ T cell epitope predicition run on all viral proteins except S (40). SARS-CoV-2 specific CD4^+^ T cells displayed significantly increased expression of activation marker following stimulation with MP_R or MP_S compared with vehicle control (DMSO) in all groups (**Figures 1A–C**). Subtraction of the DMSO background from the signal obtained with MPs allowed us to directly compare the responses obtained in the different groups of individuals and showed that OTD-CoV-2^pos^ subjects had a significantly increased frequency of virus specific CD4^+^ T cells compared with those with severe COVID-19 and with OTD-CoV-2^neg^ individuals (**Figures 1D, E**). Using a cut-off threshold based on the median values obtained after stimulation with the vehicle control (95% CI), the proportions of subjects above the threshold after stimulation with MP_R and MP_S was higher in the OTD-CoV-2^pos^ group than in severe COVID-19 patients or OTD-CoV-2^neg^ individuals (**Figures 1D, E**). MP_S induced stronger CD4^+^ T cell responses than MP_R in OTD-CoV^pos^ group only (p=0.007). When the two megapools were combined to assess the overall CD4^+^ T cell reactivity OTD-CoV-2^pos^ subjects showed a significantly higher reactivity when compared to both OTD-CoV-2^neg^ or severe COVID-19 patients (**Figure 1F**). The stimulation index, calculated as the ratio between MP and DMSO induced AIM expression, was significantly increased in OTD-CoV-2^pos^ subjects compared with patients with severe COVID-19 after stimulation with MP_S (**Supplementary Figures 4A, B**).

### Increased Frequency of SARS-CoV-2 Specific CD8 T Cells in OTD-CoV-2^pos^ Subjects

**Figure 2:**
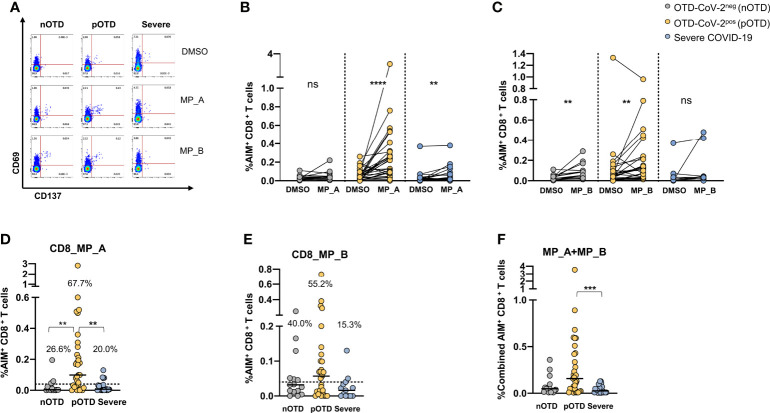
Increased SARS-CoV-2 specific CD8 T cell responses in SARS-CoV-2-infected subjects with OTD. To evaluate SARS-CoV-2-Specific CD8^+^ T cell responses, PBMC collected from 3 cohorts of subjects were stimulated for 24h with two peptide megapools: MP_A and MP_B. In all experiments, DMSO was used as negative control. **(A)** FACS plot examples of CD69 and CD137 co-expression on total CD8^+^ T cells. **(B)** Frequency of AIM+ (CD69^+^ CD137^+^) CD8^+^ T cells measured after PBMC stimulation with MP_A, **(C)** MP_B megapool, or DMSO negative control is shown for the 3 different groups. **(D)** Activation of SARS-CoV-2 specific CD8+ T cells, in the presence of MP_A and **(E)** MP_B megapools, is shown as percentage AIM+ CD8+ T cells after subtraction of the DMSO background in three cohorts of subjects. Dotted black lines indicate cut-off values for positivity. **(F)** Combined AIM^+^ CD8^+^ T cells responses to DMSO-subtracted individual peptide megapools. The non-parametric Wilcoxon matched-pairs signed-rank test was used to compare data within the same group. Statistical comparison between three groups of subjects was done by the non-parametric Kruskal-Wallis test followed by Dunn’s multiple comparisons. ns, not significant. **p < 0.01; ***p < 0.001; ****p < 0.0001.

CD8 T cells specific for SARS-CoV-2 were identified by CD69 and CD137 co-expression after stimulations with two megapools, MP_A and MP_B covering all SARS-CoV-2 proteins (40). A significantly increased expression of activation molecules was observed after stimulation with MP_A compared with DMSO in OTD-CoV-2^pos^ and severe COVID-19 patients, while stimulation with MP_B induced CD8 activation in the two OTD groups, even though lower in OTD-CoV-2^neg^ subjects, but not in severe COVID-19 patients (**Figures 2A–C**). Comparison of data obtained subtracting vehicle responses showed that CD8 MP_A was able to induce increased activation of CD8 T cells in OTD-CoV-2^pos^ subjects compared with OTD-CoV-2^neg^ subjects and severe COVID-19 patients (**Figure 2D**). No differences were observed after MP-B stimulation between the groups (**Figure 2E**). When the two megapools were combined to assess the overall CD8^+^T cell reactivity OTD-CoV-2^pos^ subjects showed a significantly higher reactivity when compared to severe COVID-19 patients (**Figure 2F**). Those comparisons were not retained when the stimulation Index was considered (**Supplementary Figures 4C, D**).

Comparison of PCR positive/Spike IgG positive (n=9) and PCR negative/Spike IgG positive pOTD patients (n=13) showed no statistically significant differences in CD4+ and CD8+ T-cell responses in the two groups (not shown).

### Increased IFNγ Production From SARS-CoV-2 Specific CD4 and CD8 T Cells in OTD-CoV-2^pos^ Subjects

**Figure 3:**
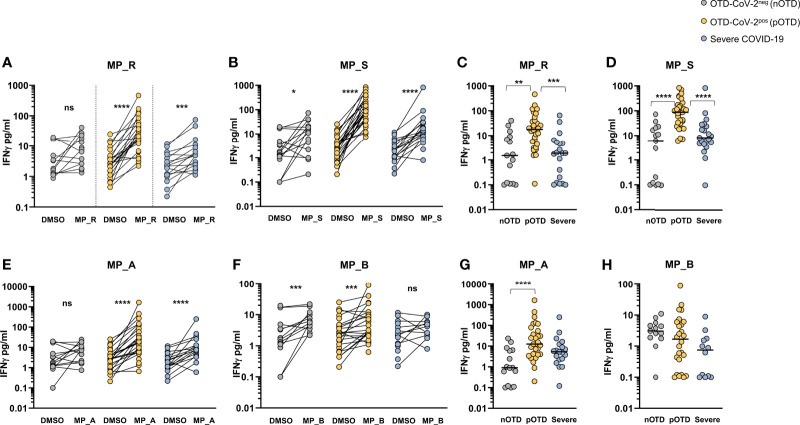
SARS-CoV-2-specific T cells from individuals with OTD produce more IFNγ than patients with severe COVID-19. **(A, B)** Amount of IFNγ (pg/mL) in the supernatants of AIM assay after PBMC stimulation with CD4-specific MP_R and MP_S peptide pools. Data are shown in comparison with the vehicle control (DMSO), per donor. **(C, D)** DMSO-subtracted IFNγ concentrations (pg/mL) after MP_R and MP_S stimulation in SARS-CoV-2 negative subjects with olfactory-taste disorder (nOTD), SARS-CoV-2 positive subjects with olfactory-taste disorder (pOTD) and severe COVID-19 patients. **(E, F)** IFNγ levels in the AIM supernatants after stimulation with CD8-specific MP_A or MP_B peptide pools. Data are shown in comparison with the vehicle control (DMSO), per donor. **(G, H)** Comparison of the background (DMSO)-subtracted IFNγ levels across the three cohorts of patients after MP_A and MP_ B stimulation. The non-parametric Wilcoxon matched-pairs signed-rank test was used to compare data within the same group. Statistical comparison between three groups of subjects was assessed by the non-parametric Kruskal-Wallis test followed by Dunn’s multiple comparisons. *p < 0.05; **p < 0.01; ***p < 0.001; ****p < 0.0001.

The T cell ability to produce IFNγ after peptide stimulation was tested in the three groups of patients. Comparison between DMSO and MP_R and MP_S stimulation showed a significant increase in IFNγ production in all groups except for MP_R induced IFNγ in the OTD-CoV-2^neg^ group (**Figures 3A, B**). Subtraction of IFNγ values obtained after stimulation with DMSO in the absence of MPs showed significantly increased levels of IFNγ production induced by MP_R and MP_S in OTD-CoV-2^pos^ compared with OTD-CoV-2^neg^ subjects and severe COVID-19 patients (**Figures 3C, D**). Similarly to CD4^+^ T-cell stimulating megapools, also CD8-MP_A induced a significant increase in IFNγ production compared with vehicle control in the severe and OTD-CoV-2^pos^ groups, but not in OTD-CoV-2^neg^ persons (**Figure 3E**). Instead, MP_B induced significant amounts of IFNγ in the two OTD groups but not in the severe COVID-19 group (**Figure 3F**). Comparison between groups after DMSO subtraction showed that MP_A was able to increase IFNγ secretion in CD8^+^ T cells from OTD-CoV-2^pos^ patients which reached statistical significance only when compared with OTD-CoV-2^neg^ subjects (**Figure 3G**). MP_B induced equivalent level of IFNγ production in all groups studied (**Figure 3H**). The data strongly suggests that SARS-CoV-2 antigen-specific T cells from paucisymptomatic individuals produce higher quantities of IFN-γ compared with T cells from severe COVID-19 patients.

### Relationship Between Anti-S1/S2 Antibodies and Increased Spike-Specific T Cell Responses in OTD SARS-CoV-2^pos^ Patients

**Figure 4:**
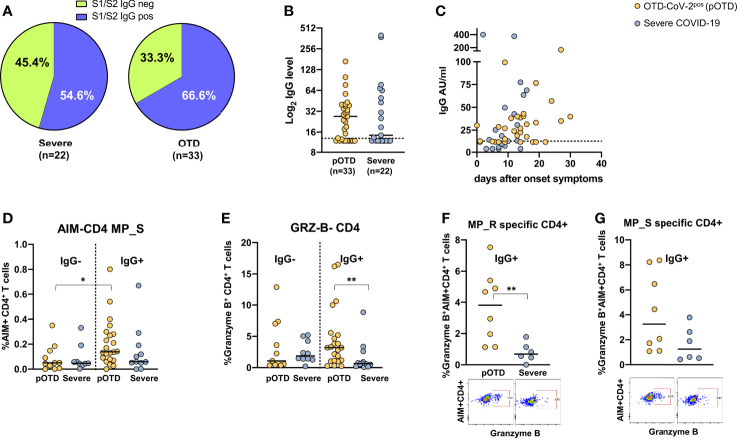
Relationship between SARS-CoV-2 T cell and antibody responses in COVID-19 individuals. **(A)** Pie chart shows the percentage of positive (blue) and negative (green) S1/S2 IgG antibody subjects in OTD-CoV-2^pos^ and severe patient cohorts. **(B)** Distribution of serum S1/S2 IgG levels in seroconverted anosmic/ageusic and severe COVID-19 individuals (IgG ≥ 13 AU/mL). **(C)** Association between time (days) from symptom onset and serum S1/S2 IgG levels. **(D)** Comparison of frequencies of AIM^+^ CD4^+^ T cells in response to the MP_S peptide pool in IgG-positive and -negative patients in OTD-CoV-2^pos^ and severe COVID-19 groups. **(E)** Frequency of CD4^+^ T cells expressing granzyme B in OTD-CoV-2^pos^ and severe COVID-19 patients stratified according to serum IgG positivity. **(F, G)** Frequency of AIM^+^ CD4 T cells expressing granzyme B in IgG- positive OTD-CoV-2^pos^ and severe COVID-19 patients following stimulation with MP_R and MP_S respectively. Mann-Whitney U test was used to compare differences between two groups. Statistical comparison between four groups of subjects was assessed by the non-parametric. *p < 0.05; **p < 0.01.

For obvious reasons, individuals with OTD and no molecular or serological evidence of current or past infection were excluded from this analysis. Detectable S1/S2 IgG antibodies were more frequently found in OTD-CoV-2^pos^ compared with hospitalized COVID-19 patients (**Figure 4A**). There were no differences in Spike-specific antibody levels between the two groups (p=0.70) (**Figure 4B**). Temporal association of appearance of anti-SARS-CoV-2 IgG and symptom onset is shown in **Figure 4C**. To assess whether the presence of S1/S2 IgG antibodies was associated with increased T cell responses, we compared spike-specific CD4^+^ T cells in IgG-positive and -negative patients in OTD-CoV-2^pos^ individuals and patients with severe COVID-19. Among the OTD-CoV-2^pos^ group, subjects who had seroconverted showed increased spike specific CD4^+^ T cells compared with those who remained S1/S2 IgG negative, while no differences were observed in hospitalized patients with severe infection (**Figure 4D**). Moreover, Granzyme B content was significantly increased in CD4^+^ T cells from serologically positive OTD patients compared with severely ill patients with positive serology (**Figure 4E**). Interestingly, antigen specific CD4^+^ T cells were significantly enriched in granzyme B content in IgG^+^ OTD-CoV-2^pos^ compared with severe patients after stimulation with MP_R (**Figure 4F**) and a similar difference, even though not statistically significant, was present after MP_S stimulation (**Figure 4G**). This trend was not present among IgG negative patients (not shown).

To understand if a positive S1/S2 IgG antibody was associated with increased germinative center (GC) response in B-cell follicles within secondary lymphoid tissues, we analyzed circulating T follicular helper cell (TFH), essential for GC formation, and frequency of plasmacells in 10 nOTD, 18 pOTD and 10 severe COVID-19 patients. The frequency of plasmacells was significantly increased in patients with severe COVID-19 compared with the other two groups (**Supplementary Figure 5A**). However, no differences were observed in proportion of TFH (**Supplementary Figure 5B**).

### Elevated Serum Inflammatory Cytokines in Severe COVID-19 Patients

**Figure 5:**
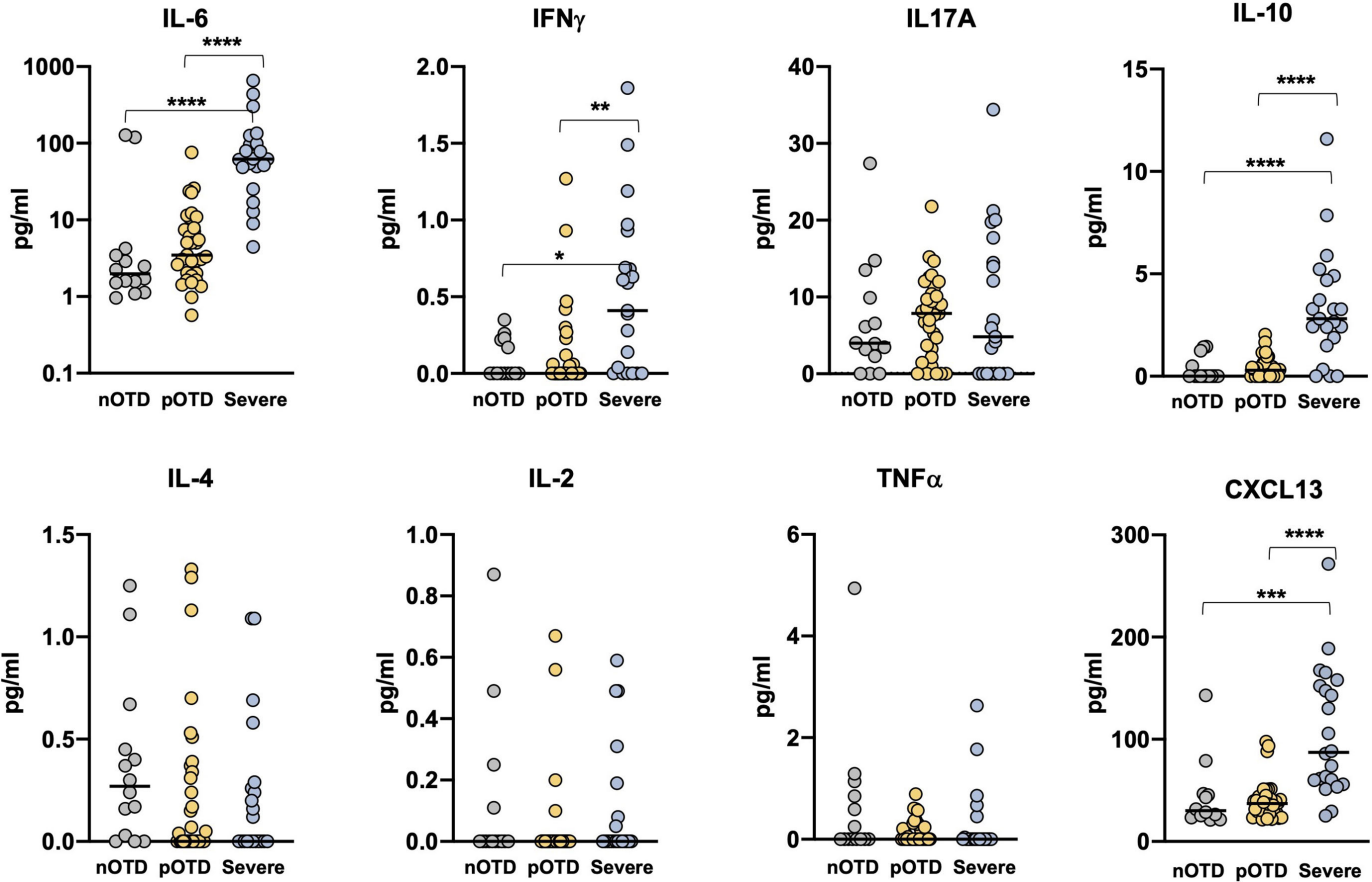
Elevated serum inflammatory cytokines in severe COVID-19 patients. Serum levels of IL-6, IFNγ, IL-17A, IL-10 (upper panels), IL-4, IL-2, TNFα and CXCL13 (lower panels) in OTD-CoV-2^neg^ subjects (nOTD), OTD-CoV-2^pos^ subjects (pOTD) and severe COVID-19 patients. Statistical comparisons between the three groups of subjects were assessed by the non-parametric Kruskal-Wallis test followed by Dunn’s multiple comparisons.**p < 0.01; ***p < 0.001; ****p < 0.0001.

We investigated Th1, Th2 and Th17 cytokine levels in the sera of OTD patients and in severe COVID-19 patients by means of cytokine bead array (CBA) technology. Increased levels of IL-6, IL-10 and IFNγ were observed only in severe patients compared with the two OTD groups, while IL-17A, TNFα, IL-4 and IL-2 concentrations were similar in the three groups (**Figure 5**). These findings are in contrast with those observed in antigen-specific T cells, where IFNγ production was instead significantly increased in the pOTD positive group compared with patients with severe COVID-19 (**Figure 3**). Moreover, we evaluated serum level of CXCL13 as an indicator of GC response in the three group of patients. CXCL13 was significantly increased in severe patients compared with pOTD and nOTD subjects (**Figure 5**).

### Hyperactivated/Exhausted T Cell Phenotype in Patients With Severe COVID-19

**Figure 6:**
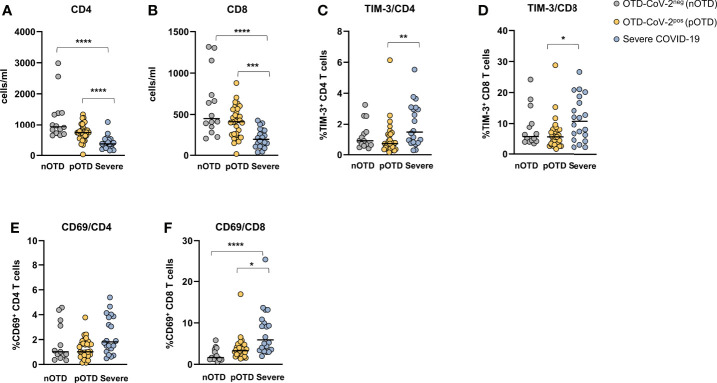
Hyperactivated/exhausted T cell phenotype in patients with severe COVID-19. Absolute numbers of circulating **(A)** CD4^+^ and **(B)** CD8^+^ T cells. **(C, D)**. Expression of the TIM-3 checkpoint molecule and **(E, F)** of the CD69 activation marker on CD4^+^ and CD8^+^ T cells. Statistical comparison between three groups of subjects was assessed by the non-parametric Kruskal-Wallis test followed by Dunn’s multiple comparisons. *p < 0.05; **p < 0.01; ***p < 0.001; ****p < 0.0001.

CD4^+^ and CD8^+^ T cell counts were significantly reduced in severe COVID-19 patients compared with the two OTD subject groups (**Figures 6A, B**). Analysis of the frequencies of T cells expressing activation (CD69) and exhaustion (TIM-3 and PD-1) molecules showed an increase of TIM-3 positive CD4^+^ and CD8^+^ T cells in severe patients compared with OTD groups (**Figures 6C, D**). Significantly higher proportions of cells expressing the CD69 early activation marker were observed only in the CD8+ T cell subset of severe patients (**Figures 6E, F**). There were no significant differences in the size of granzyme- B or PD-1 positive CD4 and CD8 T cells (not shown). No differences were observed in the proportions of central memory, effector memory and effector memory CD45RA positive T cells in the three groups, nor in their CD4^+^ or CD8^+^ T cell subsets (**Supplementary Figure 6**).

### Disorganized Immune Responses in Severe COVID-19 Patients

**Figure 7:**
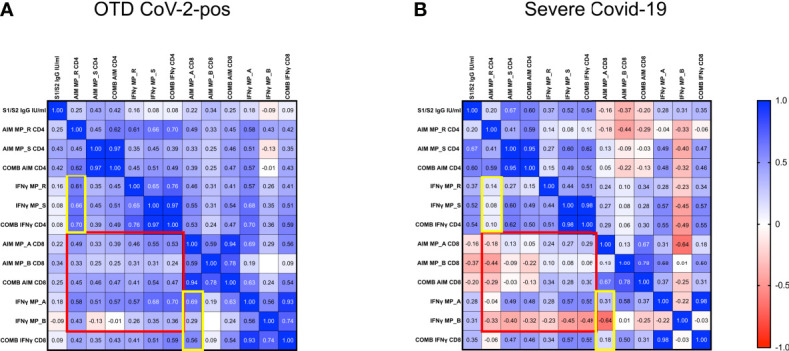
Disorganized immune responses in Severe COVID-19 patients. Spearman’s correlation matrix and clustering of 13 features in 33 OTD-CoV-2^pos^ **(A)** and 22 severe COVID-19 patients **(B)**. The scale code is shown on the right side (blue and red colors indicate positive and negative correlations, respectively).

To examine potential correlations of SARS-CoV-2 specific immune responses, we applied the Spearman rank correlation coefficients in severely ill and OTD-CoV-2^pos^ subjects. We included multiple factors, such as antigen-specific CD4^+^ and CD8^+^ T cells, combined CD4^+^ (sum of AIM induced by MP_R and MP_S) and CD8^+^ (sum of AIM induced by MP_A and MP_B) T cells, *in vitro* IFNγ secretion after stimulation with MP_R, MP_S, MP_A and MP_B, combined IFNγ secretion (sum of IFNγ induced by MP_R and MP_S or MP_A and MP_B), anti S1/S2 IgG levels. (**Figures 7A, B** and **Supplementary Table 2**).

In both OTD-CoV-2^pos^ and severe COVID-19 patients, significant correlations were observed between MP_R and MP_S induced AIM expression (CD134 and CD137) in CD4^+^ T cells. Similarly, there was a significant association between AIM expression (CD69 and CD137) in CD8^+^ T cells after MP_A and MP_B stimulation. Moreover, IFN-γ production detected in the same experiments was associated with AIM+ CD4 and CD8 T cells following MP_R and MP_A stimulation only in OTD-CoV-2^pos^ subjects (yellow box in **Figures 7A, B** and **Supplementary Table 2**).

OTD-CoV-2^pos^ patients showed a number of significant positive correlations between antigen specific CD4^+^ T cell responses and antigen specific CD8^+^ T cell responses (AIM, combined AIM, IFNγ); notably, most of these CD4/CD8 associations were not present in severe patients (red box in **Figures 7A, B**).

## Discussion

In this study, we explored T cell responses during the acute phase of the disease in OTD individuals and in hospitalized severe COVID-19 patients. We have shown that OTD subjects have increased numbers of highly functional SARS-CoV-2 specific T cells, particularly CD4^+^ T cells, compared with hospitalized patients with severe COVID-19. Several studies have identified SARS-CoV-2 specific T cells in convalescent COVID-19 individuals (24–27, 29) but only a minor proportion examined SARS-CoV-2 specific T cell responses during the early phase of infection (27, 28, 32) and virtually asymptomatic individuals. SARS-CoV-2-specific CD4^+^ T cells can be detected as early as 2–4 days after onset (27–29). Of note, the design of this study allows understanding the role of early T cell responses in the pathogenesis of the disease and, more specifically, the role of T cells in the control of infection. It has been shown that efficient SARS-CoV-2-specific CD4^+^ T cell responses are associated with milder disease progression in the early phase (27). Moreover, severe or fatal COVID-19 infection have been associated with absent or dysfunctional SARS-CoV-2-specific CD4^+^ T cell responses (27, 28, 32). In line with these observations, we showed that two thirds of paucisymptomatic subjects with OTD had detectable SARS-CoV-2 Spike specific CD4^+^ T cell responses, compared with only about a third of those with severe disease. Moreover, CD4^+^ T cell responses to non-spike peptides were also increased in OTD-CoV-2^pos^ patients compared with severe COVID-19.

Cooperation between effector T and B cells is fundamental to clear viral infections and, indeed, in SARS-CoV-2 infection a coordinated effect of both arms of adaptive immunity is associated with successful resolution of COVID-19 (24). Accordingly, our data shows that among OTD-CoV-2^pos^ subjects the presence of anti-Spike IgG was associated with a concomitant increase in Spike-specific CD4^+^ T cell responses. Evidence in support of coordinated adaptive immune responses comes from the correlograms displaying several significant positive correlations between antigen specific CD4^+^ T and CD8^+^ T cell responses which would result in an effective SARS-CoV-2 eradication. On the contrary, patients with severe disease showed dysregulated immune responses to viral peptides, resulting in a disorganized T cell specific activity, as demonstrated by the lack of synergy between CD8 and CD4 T cell responses. Our data are in agreement with a recent elegant paper which has showed that coordination between the three arms of adaptive immune response was associated with a better control of SARS-CoV-2 viral infection and that aged subjects were at risk to develop severe disease because of the lack of organized specific immune responses (27).

In line with other studies (27–29, 31), severely ill patients showed reduced frequencies of CD4^+^ T-cell specific responses and reduced IFNγ secretion in response to CD4^+^ MPs stimulation. A possible explanation for the functional impairment of antigen-specific T cells could be T cell exhaustion (41, 42). Notably, studies evaluating the immune profile of moderate/severe COVID-19 patients showed a decline in CD4^+^ and CD8^+^ T cell numbers during the acute phase of SARS-CoV-2 infection (25, 43, 44) with evidence of T cell functional exhaustion (45, 46). This is also supported by our own data showing increased TIM-3 expression on CD4^+^ and CD8^+^ T cells of patients with severe and fatal COVID-19. Indeed, terminally exhausted T cells are better defined by profoundly impaired function, rather than expression of exhaustion molecules. Upon exhaustion conditions, T cells progressively acquire inhibitory molecule expression and their secretion properties are reduced, becoming progenitor exhausted cells. When the exhaustion environment is maintained, these progenitor exhausted T cells finally differentiate into terminally exhausted T cells, which present higher levels of inhibitory molecules and null response (47). Of note, in our study, we show that IFNγ production was significantly reduced in MP-stimulated PBMC from patients with severe COVID-19 compared with those with OTD and this, together with increased expression of Tim-3 and/or PD-1 on PBMC, plausibly suggests that patients with severe COVID-19 have an increased frequency of circulating exhausted T cells compared with patients with OTD. Alternatively, it has been suggested that circulating T cells, particularly antigen-specific T cells, may be compartmentalized in the lung in patients with severe COVID-19 (48). In contrast, serum IFNγ levels were significantly increased in the severe COVID-19 group. However, it should be noted that increased levels of IFN-γ have been found during the early phase of SARS-CoV-2 infection which decreased about 10 days after clinical onset (49, 50). Of note, among our cohort of severe COVID-19 patients, those with higher levels of circulating IFNγ were recruited between 5 and 14 days from clinical onset.

CXCL13 is a proinflammatory chemokine involved in B cell activation and antibody maturation, associated with idiopathic pulmonary fibrosis (51, 52). CXCL13 has been indicated as a possible biomarker for severe COVID-19, since it has been found highly increased in severe patients, particularly in those who succumbed to the disease (53–55). In line with these studies, we observed increased serum CXCL13 in patients with severe COVID-19 compared with individuals with OTD, supporting the hypothesis that elevated levels of CXCL13 may be harmful and responsible for severe inflammation and the promotion of lung fibrosis in this setting.

Part of our study was dedicated to the analysis of T cell responses in OTD subjects and virtually no other symptoms of SARS-CoV-2 infection. They were tested for SARS-CoV-2 antibodies and viral RNA every week for 4 weeks, being persistently negative throughout the study. We surmise that these subjects had been in contact with a low viral load at the level of the upper respiratory airways that was insufficient to elicit an antiviral response or, alternatively, they were able to rapidly clear the infection. In this small group of subjects, only few developed T cell responses, but we cannot exclude a cross-reactivity with other coronavirus, as it has been shown in some healthy SARS-CoV-2 unexposed subjects (56).

Collectively, our data shows that, during the early phase of the infection, patients who will develop severe disease have reduced virus specific immune responses, that appear disorganized, potentially leading to viral escape. In contrast, OTD patients develop vigorous and coordinated virus-specific adaptive immune responses, which are likely able to block viral spread, and determine a favorable, mild course of the disease.

In conclusion, by identifying anosmic/dysgeusic individuals as a paradigm of paucisymptomatic SARS-CoV-2 infection, this study provides supporting evidence for a fundamental role of T-cell responses in limiting patients’ progression toward severe COVID-19. Lack of robust T-cell responses are instead likely to be associated with severe prognosis and outcome.

## Data Availability Statement

The raw data supporting the conclusions of this article will be made available by the authors, without undue reservation.

## Ethics Statement

The studies involving human participants were reviewed and approved by Ethical Committee of Fondazione IRCCS Policlinico San Matteo, Pavia. The patients/participants provided their written informed consent to participate in this study.

## Author Contributions

DM, SV, and MM provided substantial contribution to the conception and design of the study, acquisition, analysis and interpretation of data, and revised the manuscript critically for important intellectual content. SV and MM supervised the team, obtained funding and had leadership responsibility for the research activity planning and execution. BO, SM, and ACe contributed to acquisition, analysis, validation and interpretation of data. ACa, EM, MS, RB, and MB were responsible for data curation and were in charge of patient care. AG and AS provided peptide megapools and critically contributed to writing the manuscript. FB provided essential virological data and contributed to writing the manuscript. All authors critically read, edited and approved the final version of the manuscript.

## Funding

This work was funded by Ricerca Corrente, Ministero della Salute, Nr. RC08056520 to MM and NIH contract Nr. 75N9301900065 to AS. The authors declare that this study received additional funding support from Maurizio Traglio, Enzo Cattaneo and Alberto Borella. The funders were not involved in the study design, collection, analysis, interpretation of data, the writing of this article or the decision to submit it for publication.

## Conflict of Interest

AS is a consultant for Gritstone, Flow Pharma, Arcturus, Immunoscape, 20 CellCarta, OxfordImmunotech and Avalia.

The remaining authors declare that the research was conducted in the absence of any commercial or financial relationships that could be construed as a potential conflict of interest.

## Publisher’s Note

All claims expressed in this article are solely those of the authors and do not necessarily represent those of their affiliated organizations, or those of the publisher, the editors and the reviewers. Any product that may be evaluated in this article, or claim that may be made by its manufacturer, is not guaranteed or endorsed by the publisher.
