# Supplementary material for: High Frequencies of Functional Virus-Specific CD4+ T Cells in SARS-CoV-2 Subjects With Olfactory and Taste Disorders
Source: Front Immunol. 2021 Nov 10;12:748881. doi: 10.3389/fimmu.2021.748881 (PMC8631501; doi:10.3389/fimmu.2021.748881)
Supplement: Supplementary file 1 [file DataSheet_1.pdf]

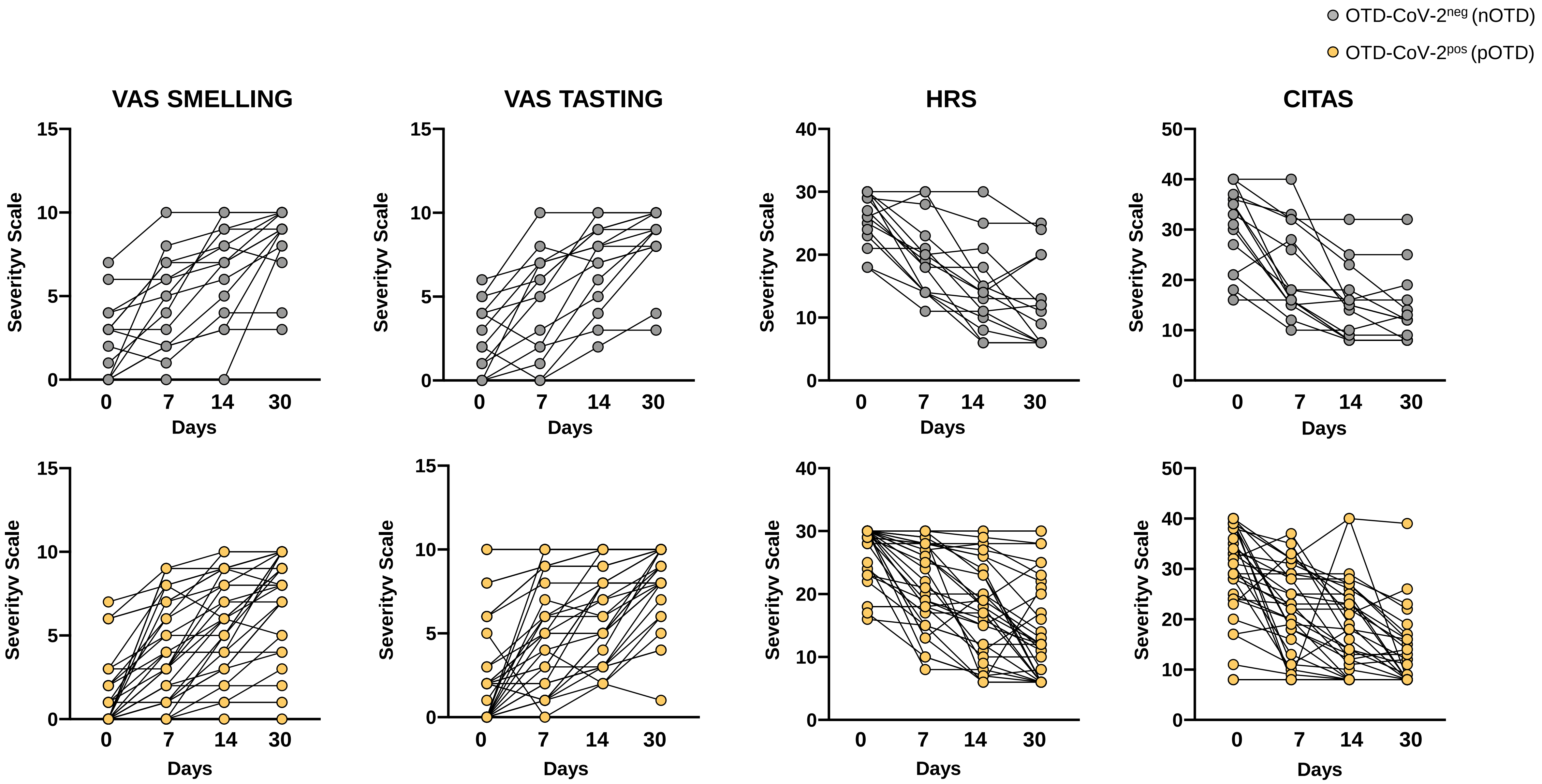

Suppl. Fig.1 Loss of smell and taste severity scored according to Visual Analogue Scale (VAS), Hyposmia Rating Scale (HRS) and Chemotherapy Induced Taste Alteration Scale (CiTAS) in OTD-CoV-2^{neg} and OTD-CoV-2^{pos} subjects.
