# Supplementary material for: High Frequencies of Functional Virus-Specific CD4+ T Cells in SARS-CoV-2 Subjects With Olfactory and Taste Disorders
Source: Front Immunol. 2021 Nov 10;12:748881. doi: 10.3389/fimmu.2021.748881 (PMC8631501; doi:10.3389/fimmu.2021.748881)
Supplement: Supplementary file 2 [file DataSheet_2.pdf]

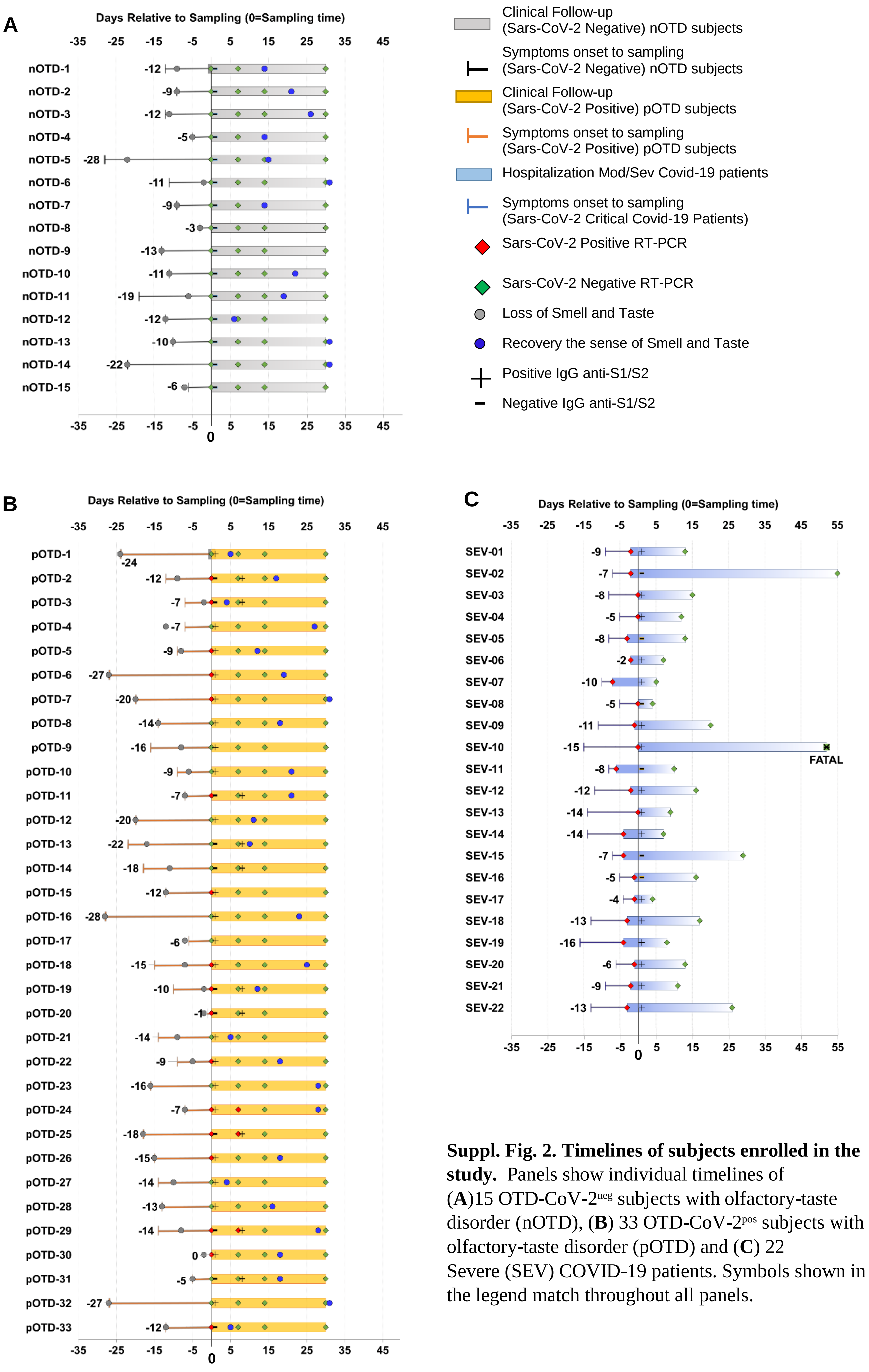

Suppl. Fig. 2. Timelines of subjects enrolled in the study. Panels show individual timelines of (**A**)15 OTD-CoV-2^{neg} subjects with olfactory-taste disorder (nOTD), (**B**) 33 OTD-CoV-2^{pos} subjects with olfactory-taste disorder (pOTD) and (**C**) 22 Severe (SEV) COVID-19 patients. Symbols shown in the legend match throughout all panels.
