# Supplementary material for: High Frequencies of Functional Virus-Specific CD4+ T Cells in SARS-CoV-2 Subjects With Olfactory and Taste Disorders
Source: Front Immunol. 2021 Nov 10;12:748881. doi: 10.3389/fimmu.2021.748881 (PMC8631501; doi:10.3389/fimmu.2021.748881)
Supplement: Supplementary file 3 [file DataSheet_3.pdf]

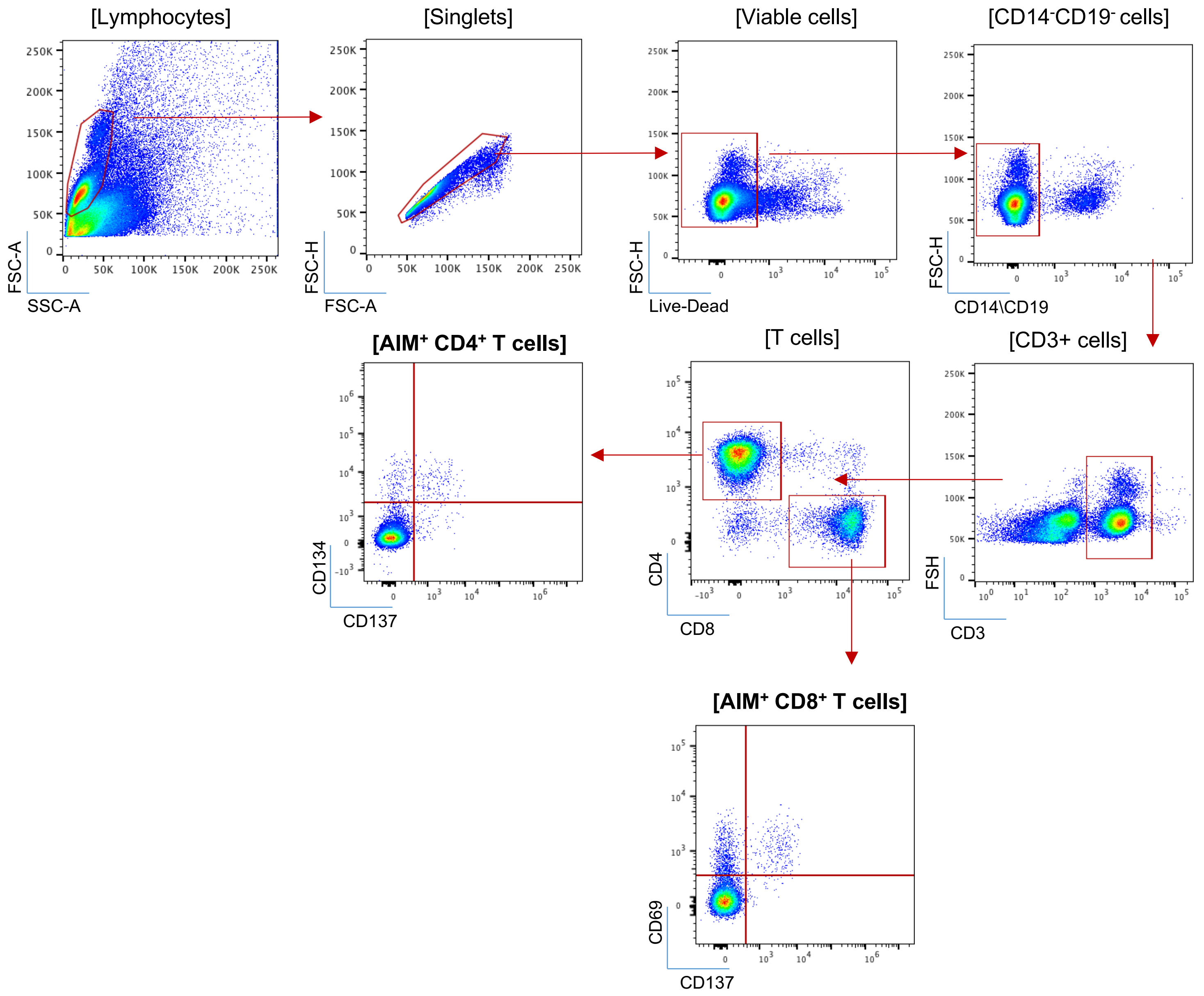

Suppl. Fig. 3. Gating strategy for identification of SARS-CoV-2 -specific CD4^{+} (CD134^{+}CD137^{+}) and CD8 (CD69+CD137^{+}) T cells.
