# Supplementary material for: High Frequencies of Functional Virus-Specific CD4+ T Cells in SARS-CoV-2 Subjects With Olfactory and Taste Disorders
Source: Front Immunol. 2021 Nov 10;12:748881. doi: 10.3389/fimmu.2021.748881 (PMC8631501; doi:10.3389/fimmu.2021.748881)
Supplement: Supplementary file 4 [file DataSheet_4.pdf]

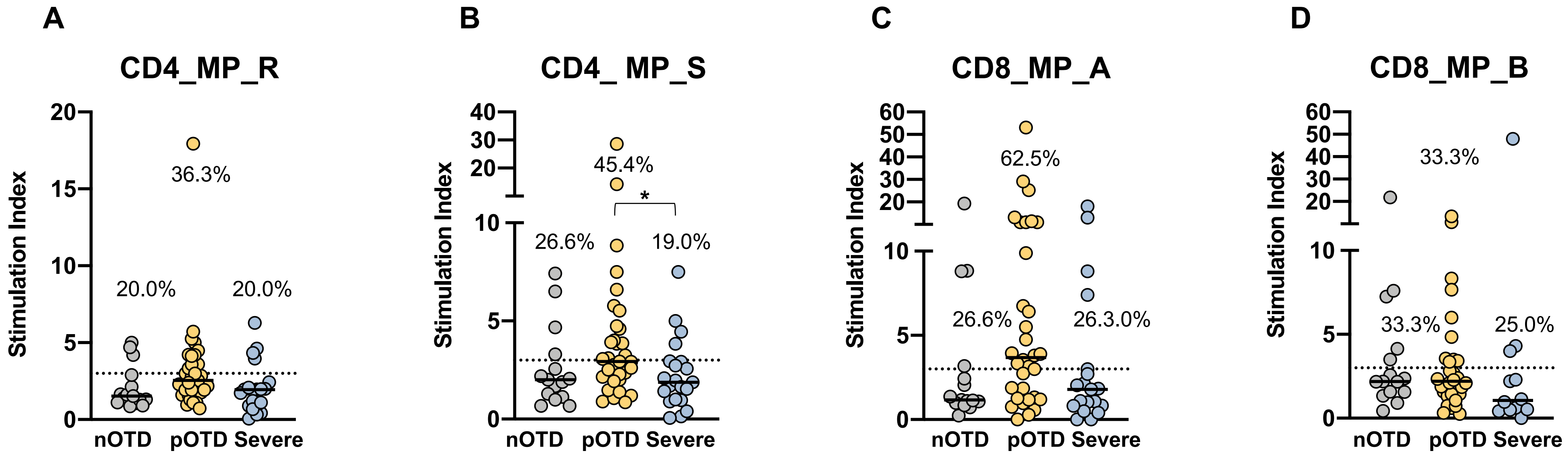

Suppl. Fig. 4 Ratio of **(A-B)** MP-specific CD4⁺ and **(C-D)** CD8⁺ T-cell activation over negative control (DMSO) activation (SI, stimulation index) in the three groups of subjetcs. Dotted black line represents the cut-off of positivity (SI>3) .
