# Supplementary material for: High Frequencies of Functional Virus-Specific CD4+ T Cells in SARS-CoV-2 Subjects With Olfactory and Taste Disorders
Source: Front Immunol. 2021 Nov 10;12:748881. doi: 10.3389/fimmu.2021.748881 (PMC8631501; doi:10.3389/fimmu.2021.748881)
Supplement: Supplementary file 5 [file DataSheet_5.pdf]

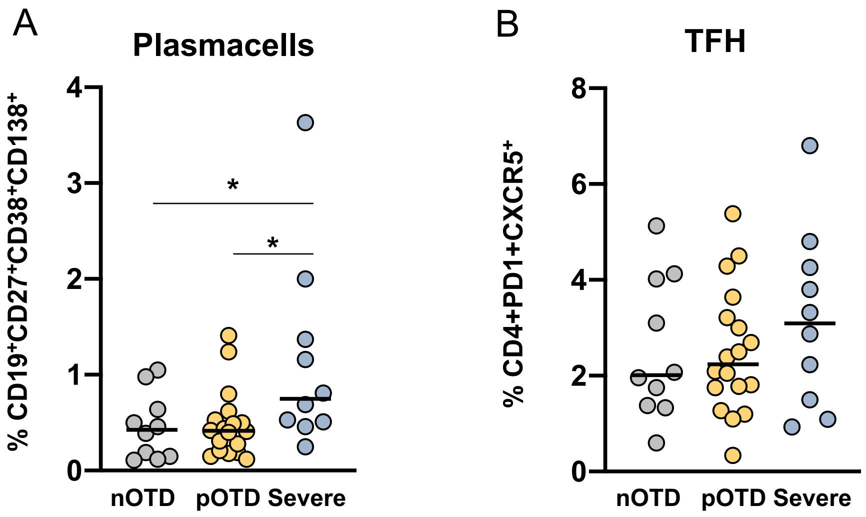

Supplementary Fig. 5. (A) Percentage of plasmacells (CD27+CD38+CD138+ B cells) in the three groups. **(B)** Frequency of circulating T follicular helper cells (Tfh), identified as PD1+CXCR5+ CD4 T cells, in SARS-CoV-2 negative subjects with olfactory-taste disorder (nOTD), SARS-CoV-2 positive subjects with olfactory-taste disorder (pOTD) and severe COVID-19 patients.
