# Supplementary material for: High Frequencies of Functional Virus-Specific CD4+ T Cells in SARS-CoV-2 Subjects With Olfactory and Taste Disorders
Source: Front Immunol. 2021 Nov 10;12:748881. doi: 10.3389/fimmu.2021.748881 (PMC8631501; doi:10.3389/fimmu.2021.748881)
Supplement: Supplementary file 6 [file DataSheet_6.pdf]

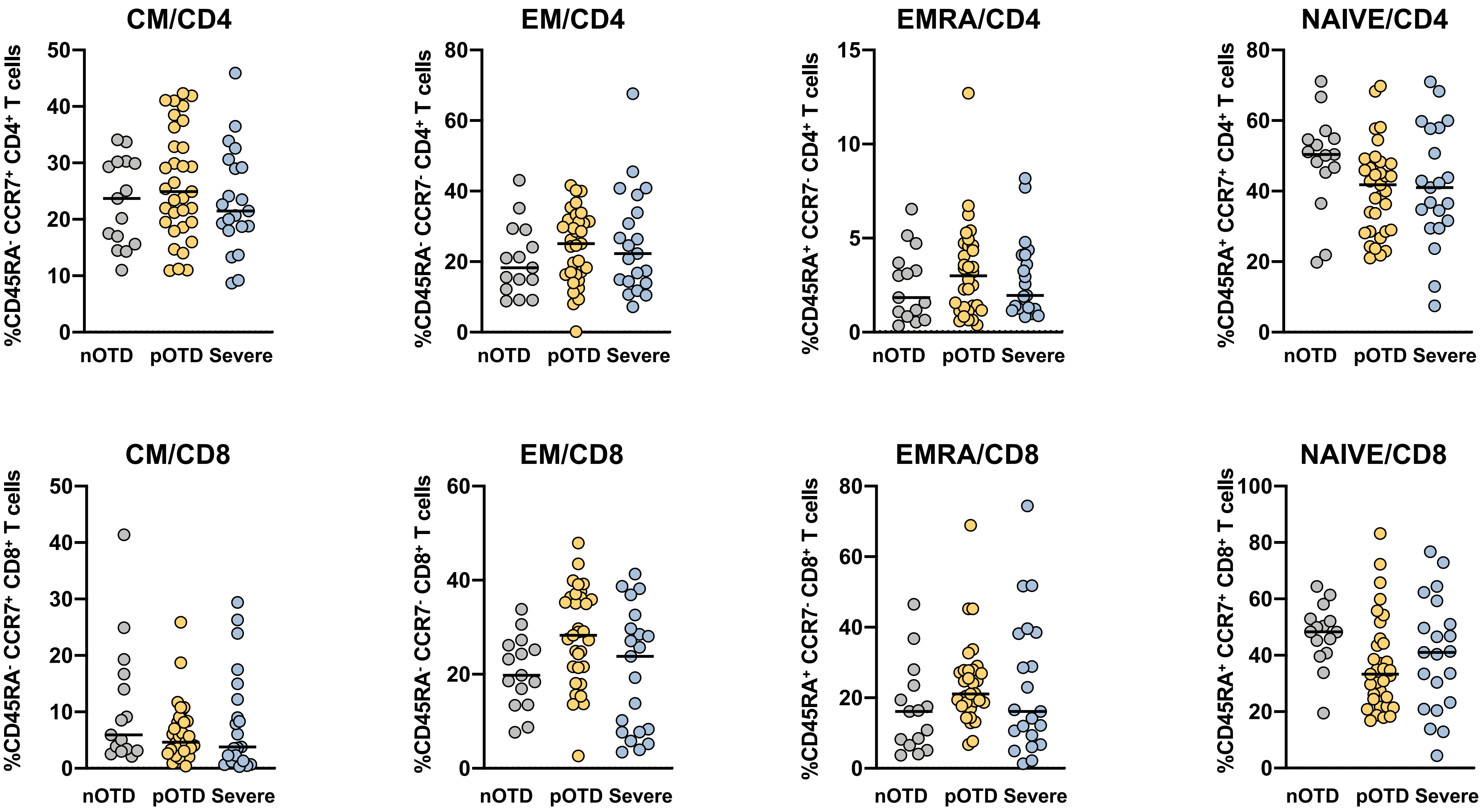

Suppl. Fig. 6 Percentage of T-cell subsets central memory (CM), effector memory (EM), effector memory CD45RA+ (EMRA) and naïve calculated out of total CD4+ (upper panel) or total CD8+ T cells (bottom panel) are shown.
