# Supplementary material for: High Frequencies of Functional Virus-Specific CD4+ T Cells in SARS-CoV-2 Subjects With Olfactory and Taste Disorders
Source: Front Immunol. 2021 Nov 10;12:748881. doi: 10.3389/fimmu.2021.748881 (PMC8631501; doi:10.3389/fimmu.2021.748881)
Supplement: Supplementary file 7 [file Table_1.docx]

| ID | Sex/Age | *Disease Severity | Fever | Cough | Dyspnea | Astenia | Diarrhea | Nausea and Vomiting | otd | bip | mip | Comorbidities |
| --- | --- | --- | --- | --- | --- | --- | --- | --- | --- | --- | --- | --- |
| SEV-01 | M/50 | Severe | Y | Y | N | N | N | N | N | Y | N | None |
| SEV-02 | M/33 | Severe | Y | Y | Y | N | N | N | N | Y | N | Diabetes Mellitus |
| SEV-03 | F/60 | Severe | Y | Y | N | N | N | N | N | N | Y | Chorea minor |
| sev-04 | M/46 | Severe | Y | Y | N | N | N | N | N | Y | N | Hypertension; Hyperlipidemia |
| sev-05 | M/60 | Severe | Y | Y | N | N | Y | Y | N | Y | N | Hypertension |
| sev-06 | F/47 | Severe | N | N | Y | N | N | N | N | Y | N | None |
| sev-07 | F/59 | Severe | N | Y | Y | Y | Y | Y | Y | N | Y | None |
| sev-08 | F/49 | Severe | Y | N | N | N | N | N | N | Y | N | None |
| sev-09 | M/53 | Severe | Y | N | Y | N | N | N | N | Y | N | Anxiety–depressive disorder |
| sev-10 | M/63 | Severe | Y | Y | Y | N | N | N | N | Y | N | Diabetes Mellitus |
| sev-11 | M/43 | Severe | Y | Y | N | N | N | N | N | N | Y | Hypertension |
| SEV-12 | M/58 | Severe | Y | N | Y | N | N | N | N | Y | N | None |
| SEV-13 | M/45 | Severe | Y | Y | Y | Y | N | N | N | Y | N | Hypertension |
| SEV-14 | M/55 | Severe | Y | Y | Y | N | N | N | N | Y | N | None |
| SEV-15 | M/66 | Severe | Y | N | N | N | N | N | N | Y | N | Diabetes mellitus; CHF |
| SEV-16 | F/47 | Severe | Y | Y | N | N | N | N | N | Y | N | None |
| SEV-17 | M/56 | Severe | Y | N | N | N | N | N | N | Y | N | None |
| SEV-18 | F/64 | Severe | Y | Y | N | N | N | N | N | Y | N | None |
| SEV-19 | M/47 | Severe | Y | Y | Y | N | Y | N | N | Y | N | None |
| SEV-20 | M/58 | Severe | N | N | Y | Y | Y | N | N | Y | N | OSAS; Hypertension |
| SEV-21 | M/60 | Severe | Y | N | Y | N | N | N | N | Y | N | POAD |
| SEV-22 | M/60 | Severe | Y | Y | Y | N | N | N | N | N | Y | COLD; Hypertension |

**Supplementary Table 1.** Demographics and clinical findings of study participants.

| ID | Sex/Age | *Disease Severity | Fever | Cough | Dyspena | Astenia | Diarrhea | Nausea or Vomiting | otd | bip | mip | Comorbidities |
| --- | --- | --- | --- | --- | --- | --- | --- | --- | --- | --- | --- | --- |
| pOTD-01 | M/61 | Mild | Y | Y | N | Y | N | Y | Y | N | N |  |
| pOTD-02 | M/70 | Mild | Y | Y | N | Y | N | Y | Y | N | N | None |
| pOTD-03 | F/57 | Mild | Y | N | N | N | Y | Y | Y | N | N | Thrombophlebitis*,* Hypertension |
| pOTD-04 | M/57 | Mild | Y | N | N | N | N | N | Y | N | N | None |
| pOTD-05 | F/48 | Mild | Y | N | N | Y | Y | N | Y | N | N | None |
| pOTD-06 | F/49 | Mild | Y | Y | N | N | N | Y | Y | N | N | None |
| pOTD-07 | F/35 | Mild | Y | N | N | N | N | Y | Y | N | N | Arrhythmia |
| pOTD-08 | F/59 | Mild | N | N | N | N | N | N | Y | N | N | Hypertension |
| pOTD-09 | F/58 | Mild | Y | Y | N | N | N | N | Y | N | N | None |
| pOTD-10 | F/47 | Mild | Y | Y | N | Y | N | N | Y | N | N | None |
| pOTD-11 | M/27 | Mild | Y | N | N | N | N | N | Y | N | N | None |
| pOTD-12 | M/59 | Mild | Y | Y | N | Y | N | Y | Y | N | N | None |
| pOTD-13 | M/64 | Mild | Y | N | N | N | N | N | Y | N | N | None |
| pOTD-14 | M/26 | Mild | Y | N | N | N | N | N | Y | N | N | None |
| pOTD-15 | F/47 | Mild | N | N | N | N | N | N | Y | N | N | None |
| pOTD-16 | M/61 | Mild | N | N | N | Y | N | N | Y | N | N | None |
| pOTD-17 | M/47 | Mild | Y | Y | N | N | N | N | Y | N | N | Hyperlipidemia |
| pOTD-18 | F/50 | Mild | Y | Y | N | N | N | Y | Y | N | N | Hypertension |
| pOTD-19 | F/51 | Mild | Y | N | N | N | N | N | Y | N | N | None |
| pOTD-20 | M/47 | Mild | N | N | N | N | N | N | Y | N | N | None |
| pOTD-21 | F/43 | Mild | Y | N | N | N | N | N | Y | N | N | None |
| pOTD-22 | F/64 | Mild | Y | N | N | N | N | N | Y | N | N | None |
| pOTD-23 | M/67 | Mild | Y | N | Y | Y | N | N | Y | N | N | Hypertension |
| pOTD-24 | F/41 | Mild | Y | Y | N | N | N | N | Y | N | N | Thalassaemia trait |
| pOTD-25 | M/30 | Mild | N | N | N | Y | N | N | Y | N | N | None |
| pOTD-26 | M/61 | Mild | Y | Y | N | Y | N | N | Y | N | N | None |
| pOTD-27 | F/41 | Mild | N | N | N | Y | N | N | Y | N | N | None |
| pOTD-28 | M/53 | Mild | N | N | N | N | N | N | Y | N | N | Hypertension |
| pOTD-29 | M/29 | Mild | Y | N | N | N | N | N | Y | N | N | None |
| pOTD-30 | F/25 | Mild | N | N | N | N | N | N | Y | N | N | None |
| pOTD-31 | F/31 | Mild | N | N | N | N | N | N | Y | N | N | Asthma |
| pOTD-32 | M/57 | Mild | Y | N | N | N | N | N | Y | N | N | None |
| pOTD-33 | M/23 | Mild | N | N | N | N | N | N | Y | N | N | None |

| ID | Sex/Age | *Disease Severity | Fever | Cough | Dyspena | Astenia | Diarrhea | Nausea or Vomiting | otd | bip | mip | Comorbidities |
| --- | --- | --- | --- | --- | --- | --- | --- | --- | --- | --- | --- | --- |
| nOTD-01 | 39/f | - | Y | Y | N | Y | N | Y | Y | N | N | CMT |
| nOTD -02 | 55/F | - | N | N | N | N | N | N | Y | N | N | None |
| nOTD -03 | 30/F | - | Y | Y | N | Y | N | Y | Y | N | N | Fuchs’ Dystrophy |
| nOTD -04 | 61/F | - | N | N | N | N | N | N | Y | N | N | None |
| nOTD -05 | 36/F | - | Y | N | N | N | N | N | Y | N | N | GERD |
| nOTD 06 | 59/M | - | N | Y | N | Y | N | Y | Y | N | N | None |
| nOTD -07 | 34/F | - | Y | N | N | N | N | N | Y | N | N | None |
| nOTD -08 | 35/M | - | N | N | N | N | N | N | Y | N | N | None |
| nOTD-09 | 45/M | - | Y | N | N | N | N | Y | Y | N | N | None |
| nOTD -10 | 46/F | - | Y | Y | Y | N | N | Y | Y | N | N | None |
| nOTD -11 | 36/M | - | Y | Y | N | N | N | Y | Y | N | N | None |
| nOTD -12 | 39/F | - | N | N | N | N | N | N | Y | N | N | None |
| nOTD -13 | 48/F | - | N | N | N | N | N | N | Y | N | N | None |
| nOTD-14 | F/58 | - | N | Y | N | N | N | N | Y | N | N | None |
| nOTD-15 | M/26 | - | N | N | N | N | N | N | Y | N | N | None |

***Mild**: Individuals who have any of the various signs and symptoms of COVID-19 but who do not have shortness of breath, dyspnea, or abnormal chest imaging; **Severe:** Individuals who have SpO2 <94% on room air at sea level, a ratio of arterial partial pressure of oxygen to fraction of inspired oxygen (PaO2/FiO2) <300 mm Hg, respiratory frequency >30 breaths/min, or lung infiltrates >50%. Requiring oxygen supplemntation FiO2>0.5, high flow oxygen and/or medical ventilation [[COVID-19 Treatment Guidelines (nih.gov)](https://www.covid19treatmentguidelines.nih.gov/), April, 2021 Update]; BIP: Bilateral Interstitial pneumonia; MIP: Monolateral Interstitial pneumonia; CHF: Congestive Heart Faliure; OSAS: Obstructive Sleep Apnea Syndrome; POAD: Peripheral Obliterative Arteriopathy Disease; COLD: Chronic Obstructive Lung Disease; CMT: Charcot-Marie-Tooth; GERD: Gastroesophageal reflux disease.
